# Long-term pioglitazone use in MASLD patients: insights from a multicentric preliminary study

**DOI:** 10.1016/j.clinsp.2025.100737

**Published:** 2025-08-21

**Authors:** Isabel Veloso Alves Pereira, Ana Beatriz Souza de Oliveira, Patricia Momoyo Yoshimura Zitelli, Lais Arrivabene Barbieri, Ana Carolina Cardoso, Mísia Joyner de Sousa Dias Monteiro, Juliana Souza de Oliveira, José Tadeu Stefano, Renato Altikes, Ana Luiza Gomes Reis, Cláudio de Figueiredo Mendes, Claudia Couto, Nathalie C. Leite, Cristiane A. Villela-Nogueira, Claudia P. Oliveira, Mário G Pessoa

**Affiliations:** a Divisão de Gastroenterologia e Hepatologia, Hospital das Clínicas HCFMUSP (LIM-07), Departamento de Gastroenterologia e Nutrologia, Faculdade de Medicina, Universidade de São Paulo, São Paulo, SP, Brazil; b Universidade Federal de Minas Gerais, Belo Horizonte, MG, Brasil; c Hospital Universitário Clementino Fraga Filho, Faculdade de Medicina, Universidade Federal do Rio de Janeiro, Rio de Janeiro, RJ, Brazil

**Keywords:** MASLD, MASH, Pioglitazone

## Abstract

**Background and Aims:**

This multi-center retrospective study evaluated the long-term effects of pioglitazone therapy on liver stiffness, hepatic steatosis, and other non-invasive biomarkers in patients with Metabolic Dysfunction-Associated Steatotic Liver Disease (MASLD).

**Methods:**

A total of 65 patients from three Brazilian public hospitals treated with pioglitazone at doses of 30‒45 mg/day up to 10-years were retrospectively analyzed. Hepatic parameters, including Vibration-Controlled Transient Elastography (VCTE), Controlled Attenuation Parameter (CAP), and FibroScan-AST score, were evaluated before and after treatment. Patients were stratified into two groups based on treatment duration: 1‒3 years and 4‒10 years.

**Results:**

Significant reductions in Alanine Aminotransferase (ALT) and Gamma-Glutamyl Transferase (GGT) levels were observed in both treatment groups, indicating improvement in liver enzyme profiles. A significant decrease in CAP levels was observed only in the 4‒10 year group (*p* = 0.002), suggesting a reduction in liver steatosis. Improvement in FAST™ scores was observed in both groups (1‒3 years, *p* = 0.042; 4‒10 years, *p* = 0.012). In logistic regression analysis, dyslipidemia was associated with a non-significant trend toward higher odds of liver stiffness reduction (adjusted OR = 1.92, 95 % CI 0.58‒6.45, *p* = 0.284).

**Conclusions:**

These findings highlight both the metabolic and hepatic benefits of long-term pioglitazone therapy. They reinforce its potential as a cost-effective and accessible treatment option for MASLD, particularly in resource-limited settings where newer therapeutic alternatives are unavailable.

## Highlights

• Long-term pioglitazone therapy significantly reduces hepatic fat accumulation, as evidenced by reductions in CAP and FAST™ scores.
• Pioglitazone demonstrated dual benefits by improving both liver parameters and glycemic control in MASLD patients.
• Non-invasive tools, such as VCTE and FAST™ score, are valuable for monitoring treatment responses and reducing the need for liver biopsies.

## Introduction and objectives

**Fig. 1:**
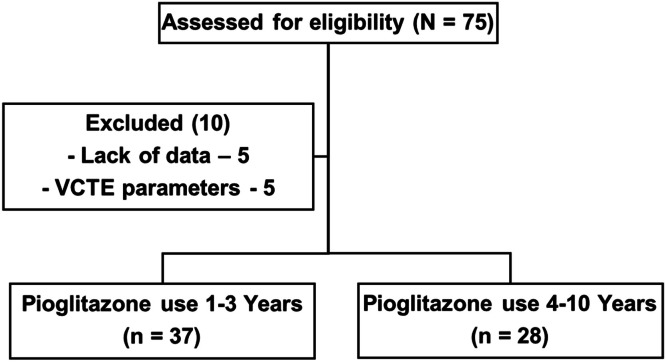
Flow diagram.

Pioglitazone, a thiazolidinedione, is a Peroxisome Proliferator-Activated Receptor (PPAR) agonist. It changes lipid distribution, increases insulin sensitivity by enhancing adiponectin levels, and contributes to anti-inflammatory and anti-fibrotic effects.^1^^,^^2^ In light of this data, various studies have provided compelling evidence that Pioglitazone could improve histological liver parameters and effectively ameliorate Metabolic Dysfunction-Associated Steatotic Liver Disease (MASLD).^3^^,^^4^ Several prospective studies have shown that patients with or without Type 2 Diabetes Mellitus (T2DM) who received 30 mg or 45 mg of Pioglitazone daily had better outcomes than those who received a placebo, including MASH resolution, fibrosis improvement, and even reversal of fibrosis (Fig. 1).^5^

MASLD is recognized as a global health problem, due to its rising prevalence, affecting about 30 % of Western countries’ population. In certain regions, this proportion is notably higher, reaching 44.37 % in Latin America (with a range of 31 % to 59 %).^6^ Its trajectory parallels the growing epidemic of obesity, with MASLD projected to become the leading cause of liver transplantation in the coming years, underscoring its potential severity and impact on public health.^7^

Given the high and increasing prevalence of MASLD, performing liver biopsies on all patients would be impractical and inadequate, potentially leading to unnecessary invasive procedures. Therefore, a population-based approach should prioritize biopsies only in selected patients. Nevertheless, it is necessary to identify patients with higher risks of progressing to advanced fibrosis, as it changes the periodicity of surveillance of outcomes and highlights the patients who would benefit most from new pharmacological interventions.^8^

Vibration-Controlled Transient Elastography (VCTE) is an ultrasound-based elastography technique validated in several studies with high reliability and reproducibility.^9^^,^^10^ Stratifying patients based on their VCTE scores also holds promise for predicting liver-related events. A large cohort study concluded that VCTE is a suitable alternative to liver biopsy in routine practice and even in phase 2b and 4 clinical trials to assess improvement with proposed treatments.^11^

Another promising noninvasive tool, the FAST™ score, integrates FibroScan® parameters Liver Stiffness Measurement (LSM), Controlled Attenuation Parameter (CAP), and Aspartate Aminotransferase (AST).^12^ It has shown good diagnostic accuracy for at-risk MASH (Area-Under-the-Receiver-Operating-Characteristic [AUROC = 0.80]) in European and North American cohorts, and a Brazilian MASLD population.^13^^,^^14^ Furthermore, recent data indicate that a reduction in the FAST™ score of >0.22 points is significantly associated with MASH resolution without worsening of fibrosis, supporting its use as a potential primary endpoint in trials assessing treatment efficacy.^15^

Therefore, this research aims to evaluate whether sustained Pioglitazone use over 1 to 10 years can improve the VCTE parameters (liver stiffness and CAP) and the FAST™ score in individuals with MASLD.

## Methods

### Design and subjects

This retrospective multicenter study analyzed patients diagnosed with Metabolic Dysfunction-Associated Steatotic Liver Disease (MASLD) who received treatment with Pioglitazone (30–45 mg) at three public hospitals in the southwestern region of Brazil. To qualify for inclusion, patients need to be at least 18-years-old, have used Pioglitazone for one year, and have both pre- and post-treatment transient elastography evaluations (VCTE) at the end of follow-up, along with complete medical records. Patients were excluded if they lacked sufficient data or if the pre-treatment VCTE evaluation was performed more than eight months before starting Pioglitazone. Additional exclusion criteria included conditions that could affect elastography accuracy, such as severe cholestasis (total bilirubin > 5 mg/dL), liver enzyme levels > 5 times the upper limit of normal, advanced ascites, acute inflammatory or infectious conditions (e.g., cholangitis), recent food intake (< 2–4 h prior), liver congestion (e.g., heart failure), focal liver lesions in the measurement area, or technical issues due to poor patient cooperation. This study was reported in accordance with the Strengthening the Reporting of Observational Studies in Epidemiology (STROBE) guidelines.

Patients were prescribed Pioglitazone for MASLD associated with T2DM or in cases where liver biopsy results indicated fibrosis graded as ≥F2. To thoroughly investigate the impact of Pioglitazone duration on clinical outcomes in MASLD patients, the authors categorized the data into two groups: those using the medication for up to three years and those using it for 4 to 10 years. This classification was essential for identifying any variations in treatment response over time. The primary outcome of this study was the change in the FAST™ score from baseline to the end of follow-up, used as a non-invasive marker to assess hepatic improvement in patients with MASLD undergoing long-term pioglitazone therapy.

### Outcome measures

All patients' demographic, anthropometric, and clinical data were gathered, including age, gender, height, weight, Body Mass Index (BMI), presence of T2DM, dyslipidemia, Systemic Arterial Hypertension (SAH), FIB-4 and FAST™ score, use of losartan and statins. Serum biochemistry tests conducted in all patients included platelets, Alanine Aminotransferase (ALT), Aspartate Aminotransferase (AST), Gamma-Glutamyl Transferase (GGT). Total cholesterol, HDL, and LDL. Liver Stiffness Measurement (LSM) by Vibration-Controlled Transient Elastography (VCTE) and CAP were both measured using FibroScan**®** machines equipped with both M and XL probes (Echosens, Paris, France) by nurses or physicians trained. The measurements were validated with an Interquartile Range (IQR) of <30 %.

### Statistical analysis

The data were analyzed using JAMOVI® software version 2.5 and GraphPad Prism 10.^16^^,^^17^ Qualitative variables, expressed as percentages, were subjected to the Chi-Square test to verify the existence of significant associations between categories. Quantitative variables were described using median (IQR) and differences between groups were evaluated using the Wilcoxon test (for paired samples). Logistic regression analysis was performed to evaluate the association between clinical factors and LSM reduction, presenting adjusted Odds Ratios (ORs) with 95 % Confidence Intervals (95 % CIs). Although this was a retrospective analysis, the authors performed a post-hoc sample size estimation to validate the statistical power of the primary outcome (FAST™ score reduction). Considering a mean paired difference of 0.12 and a standard deviation of 0.36, a sample size of 73 patients would be required to detect this effect with 80 % power and α = 0.05. The present study included 65 patients, which approaches the estimated requirement, supporting the reliability of these findings while acknowledging this as a limitation.

## Results

A total of 65 patients were included in the study. Of these, 39 (60 %) were female, with a mean age of 58±10.23 years. At baseline, 50 (74 %) had SAH, 48 (74 %) had T2DM, and 47 (69 %) had dyslipidemia. Among the study participants, 10 patients (15.4 %) were using insulin, 55 (84.6 %) were on metformin, and 8 (12.3 %) were receiving GLP-1 receptor analogues during the treatment period. Additionally, 31 (46 %) were treated with losartan and 41 (60 %) were treated with statins. Pioglitazone use was well tolerated, and its discontinuation was due to the medication's unavailability through the popular pharmacy program, rather than arising from adverse side effects. This distinction is vital for accurately analyzing the results, ensuring that conclusions rest firmly on the medication’s efficacy and tolerability, independent of external supply issues. Table 1 displays the anthropometric and laboratory data at baseline and at the end of the study.

**Table 1:** Clinical and laboratory characteristics of MASLD patients treated with pioglitazone over different durations (1–3 years vs. 4–10 years).

	Pioglitazone 1‒3 years (*n* = 37)			Pioglitazone 4‒10 years (*n* = 28)		
Variable	Pre‒treatment	Post-treatment	p	Pre-treatment	Post-treatment	p
Age (years)	59 (16; 37‒76)	61 (14; 39‒78)	0.05	61 (10.25; 18‒73)	66.50 (10.5; 26‒79)	0.05
Weight (Kg)	84 (14.40; 56.50‒120)	89.40 (25.97; 63‒103)	NS	82.5 (19.58; 60‒99.4)	83.50 (7.5; 76‒91)	NS
BMI (Kg/m^2^)	30.30 (5.60; 23.30‒44.07)	30 (8.40; 22.80‒44.80)	NS	31.45 (5.63; 24‒40.33)	30.93 (3.03; 23.10‒40.77)	NS
Platelets (10^9^ mm^3^)	216 (107; 79‒377)	228 (103; 108‒368)	NS	202 (104.5; 83‒359)	239 (124.25; 98‒660)	NS
ALT (U/I)	41 (29; 11‒211)	34 (28; 12‒181)	**0.028**	40.5 (26.25; 21‒117)	31.5 (20.5; 13‒77)	**0.012**
AST (U/I)	37 (25; 17‒102)	31 (19; 11‒136)	NS	32 (17.5; 15‒79)	26.5 (22; 13‒92)	NS
GGT (U/I)	79 (78; 11‒647)	41 (65; 15‒1001)	**0.041**	55 (62.25; 17‒452)	80 (119.5; 10‒427)	NS
LDL (mg/dL)	93 (35; 26‒154)	81 (36.75; 34‒161)	NS	84 (40; 48‒162)	88.5 (31.25; 9‒138)	NS
Total Cholesterol (mg/dL)	173 (35; 92‒265)	164.50 (48.25; 90‒296)	NS	170.5 (38.25; 109‒220)	160.50 (41.50; 85‒238)	NS
HDL (mg/dL)	45 (19; 30‒101)	44 (16.25; 25‒104)	NS	45 (14; 31‒77)	53.50 (10.75; 32‒68)	NS
TG (mg/dL)	129 (98; 64‒414)	150.50 (86; 54‒381)	NS	125 (72.5; 45‒345)	113 (47; 36‒212)	NS
HbA1c ( %)	6.65 (1.77; 5.20‒13.80)	7.05 (2.10; 4.70‒13)	NS	6.85 (1.85; 4.8‒13.4)	6.25 (1.08; 4.40‒8.20)	**0.040**
Serum Glucose (mg/dL)	118 (53; 78‒226)	113 (59.50; 86‒296)	NS	119 (38.5; 83‒314)	101 (19.75; 72‒168)	**0.051**
LSM (Kpa)	8.10 (6.10; 2.80‒35.30)	6.90 (8.60; 3.70‒50.50)	NS	10.70 (7.05; 5.6‒48)	9.55 (10.53; 4.50‒50.40)	NS
CAP	323 (72; 180‒400)	307 (49; 151‒400)		330.50 (42.5; 160‒400)	299 (62.75; 174‒400)	**0.002**
FIB-4	1.60 (1.40; 0.50‒8.60)	1.60 (1.00; 0.50‒4.90)	NS	1.35 (1.35; 0.5‒4.1)	1.20 (1.05; 0.6‒4.5)	NS
FAST™ SCORE	0.44 (0.36; 0.02‒0.90)	0.32 (0.29; 0.01‒0.94)	**0.042**	0.49 (0.51; 0.06‒0.91)	0.30 (0.36; 0.06‒0.78)	**0.012**

This table presents clinical and laboratory data for patients with Metabolic Dysfunction-Associated Steatotic Liver Disease (MASLD) treated with Pioglitazone for 1–3 years (*n* = 37) and 4–10 years (*n* = 28). Data are expressed as median (interquartile range; range), and pre-treatment and post-treatment values are compared using the Wilcoxon Test, with p-values indicating the significance of changes. Significant p-values (< 0.05) are highlighted, indicating statistically meaningful changes between pre- and post-treatment assessments.

NS, Non-Significant; BMI, Body Mass Index, calculated as weight in kilograms divided by the square of height in meters; ALT, Alanine Aminotransferase; AST, Aspartate Aminotransferase; GGT, Gamma-Glutamyl Transferase; LDL, Low-Density Lipoprotein cholesterol; HDL, High-Density Lipoprotein Cholesterol; TG, Triglycerides; HbA1c, Glycated Hemoglobin; LSM, Liver Stiffness; CAP, Controlled Attenuation Parameter; FIB-4, Fibrosis-4 Index; FAST™ SCORE, FibroScan-AST score.

**Fig. 2:**
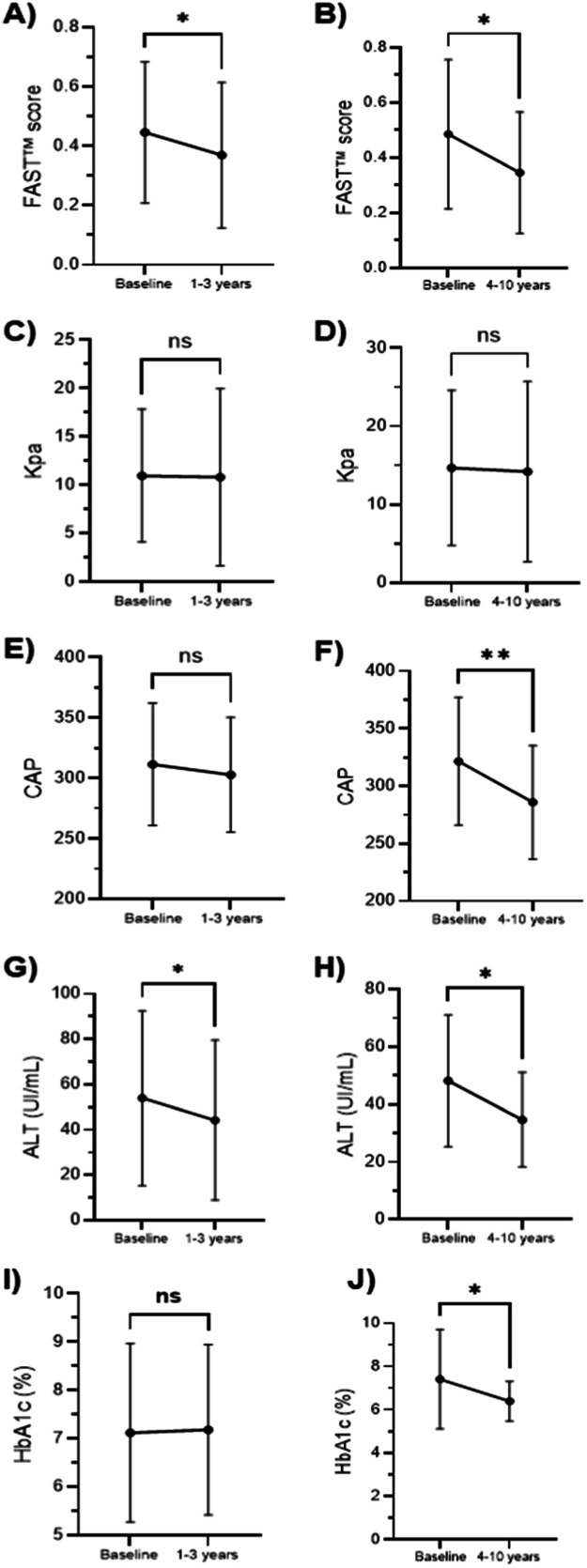
Parameters affected by Pioglitazone use. A and B show the FAST™ score at baseline and over time (A: 1–3 years, B: 4–10 years), with significant reductions indicated (*p < 0.05). C and D display Liver Stiffness (kPa), showing no significant changes over time (ns = not significant). Panels E and F represent Controlled Attenuation Parameter (CAP), reflecting liver fat levels. While panel E (1–3 years) shows no significant difference, panel F (4–10 years) shows a significant reduction (**p < 0.01). G and H illustrate Alanine Aminotransferase (ALT) levels, with significant reductions observed in both timeframes (*p < 0.05). Panels I and J show HbA1c ( %) changes, with no significant change in the 1–3 years group (I) but a significant reduction (*p < 0.05) in the 4–10 years group (J).

The longitudinal analysis of health parameters from baseline through 1‒3 years and 4‒10 years reveals significant changes in key biomarkers, indicating improvements in liver enzymes, hepatic steatosis, and glycemic control over time (Fig. 2). A significant reduction in FAST™ score was observed between baseline and the 1–3 years follow-up period (*p* = 0.042) and was further pronounced in 4‒10 years period (*p* = 0.012). Measurements of LSM (Kpa) at baseline and at the end of follow-up did not show a significant difference, which probably reflects the variability of LSM changes between the patients. However, the authors observed a reduced or maintained LSM (Kpa) in 61.5 % (*n* = 40), varying from 0 to 15.5 (0‒72.8 %). CAP parameters showed a significant reduction only between baseline and the 4–10 year follow-up period (*p* = 0.002), suggesting a decrease in hepatic fat accumulation over the longer term. ALT was reduced in both time groups, demonstrating an improvement in liver enzymes. In addition, an improvement in glycemic control was observed in the 4‒10 year period, highlighting Pioglitazone's dual benefit in addressing metabolic and hepatic dysfunctions.

**Fig. 3:**
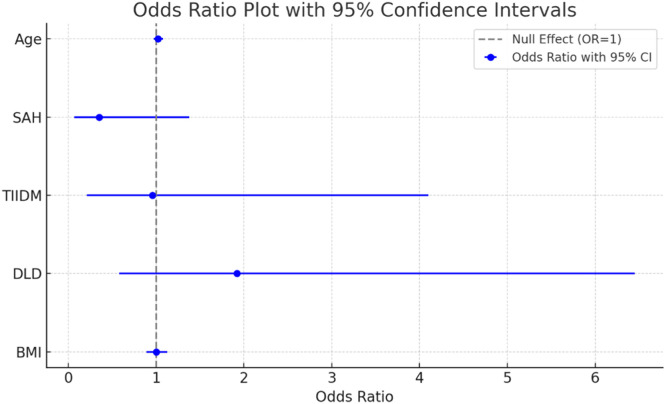
Logistic regression – LSM reduction. SAH, Systemic Arterial Hypertension; T2DM, Type 2 Diabetes Mellitus; DLD, Dyslipidemia; BMI, Body Mass Index.

The logistic regression analysis investigated the association between clinical factors and LSM reduction (Fig. 3). Although no predictors were statistically significant, dyslipidemia exhibited a trend toward higher odds of LSM reduction (adjusted OR = 1.92, 95 % CI 0.58–6.45, *p* = 0.284). The model demonstrated fair discrimination (C-statistic = 0.652) and good calibration (Hosmer-Lemeshow *p* = 0.592).

The “rule in/rule out” approach for the FAST™ score (≤ 0.35) described by Newsome et al.,^12^ to stratify patients based on their likelihood of having significant liver outcomes was applied to better understand the Pioglitazone use and improvement in liver function. A 25 % reduction in the proportion of at-risk patients and an 8 % (*n* = 5) progression was observed (Table 2).

**Table 2:** Rule In/Rule out FAST score.

		Pioglitazone 1‒3 years (*n* = 32)	Pioglitazone 4‒10 years (*n* = 25)	Total (*n* = 60)
**Rule In/Out FAST score ≤0.35**	In-In	12 (38 %)	11 (39 %)	23 (38 %)
	In-Out	7 (22 %)	8 (29 %)	15 (25 %)
	Out-Out	10 (30 %)	7 (25 %)	17 (28 %)
	Out-In	3 (9 %)	2 (7 %)	5 (8 %)

## Discussion

In this retrospective study, the authors gathered data from 65 patients with MASH receiving pioglitazone treatment for 1‒10 years, from three public hospitals in Brazil. The authors compared their parameters on VCTE before and after the treatment to assess whether liver stiffness could be improved. Examination of laboratory and clinical data was done as well. The authors found a significant improvement in aminotransferases and GGT, as well as elastography parameters, mainly for steatosis evaluated by CAP. This is the first Brazilian study that evaluates the effect of Pioglitazone in MASLD patients.

Cusi et al. demonstrated that pioglitazone therapy led to a 47 % resolution of NASH in patients with and without diabetes, with concurrent reductions in liver enzymes, suggesting improved hepatic inflammation and injury markers.^18^ While it is not reliable to use enzyme values alone to infer the severity of liver disease, ALT levels have been shown to decrease in patients who experience treatment-related improvement or resolution of histological MASH.^19^^,^^20^ Similarly, a recent study in the pediatric population with MASLD showed that variations in serum ALT and GGT were strongly associated with changes in liver histology and proposed that these markers could be useful as an indicator of histologic response.^21^

Although many other studies established the role of pioglitazone in enhancing MASH and even fibrosis, its use is still off-label.^5^ Until now, the only US Food and Drug Administration (FDA) approved drug to treat patients with MASH and moderate to advanced liver fibrosis is Resmetirom.^22^

Especially for more vulnerable countries, such as Brazil, it would be prohibitively expensive to provide this drug to treat all patients with MASH, limiting access to health and exacerbating disparities. On that account, the motivation for this study arises from the urgent need to find accessible and effective treatments for MASH. Given the widespread impact of MAFLD and MASH, it is imperative to explore alternative therapeutic options that are both affordable and effective. An off-label use of pioglitazone presents a promising candidate due to its established safety profile and recurrent evidence suggesting potential benefits for treating MASH. By investigating the efficacy of pioglitazone in this context, the authors aim to reinforce it as an accessible treatment option, thereby improving patient outcomes and reducing the financial burden associated with managing MAFLD.

Overall, while the absence of histology data is a limitation, the observed biochemical changes and elastography parameters in the present study provide valuable insights into the potential efficacy of Pioglitazone in improving liver disease biomarkers.

The high prevalence of T2DM among the study population (78 %) underscores the overlap between MASLD and metabolic comorbidities. Literature consistently demonstrates that T2DM accelerates fibrosis progression and increases the risk of adverse hepatic outcomes in MASLD. For instance, studies by Bril et al. (2018) and Cusi et al. (2016) have highlighted the importance of addressing insulin resistance in this population, positioning pioglitazone as a logical therapeutic choice.^18^^,^^23^

In addition to its hepatic benefits, pioglitazone’s effects on glycemic control were observed in the present study, particularly among long-term users. These findings are consistent with the dual action of pioglitazone in improving peripheral insulin sensitivity and reducing hepatic fat accumulation. Such dual benefits are critical in managing the complex interplay between T2DM and MASLD.

Without a doubt, the overall changes in scores have better predictive abilities than the changes in individual biomarkers alone. In this context, the authors evaluated changes in FAST, in CAP, and in LSM. The reduction in FAST™ scores observed highlights the utility of this composite marker in assessing treatment responses. Studies have validated FAST™ as a reliable tool for identifying patients with at-risk MASH and tracking changes in liver health during therapeutic interventions. For example, a recent trial evaluating semaglutide reported significant correlations between FAST™ score improvements and histological resolution of MASH, reinforcing its clinical relevance.^15^ The significant reduction in CAP values, particularly in long-term pioglitazone users, suggests a decrease in hepatic fat accumulation. This aligns with findings from the PIVENS trial, which showed that pioglitazone improved hepatic steatosis as assessed by histology. CAP, as a non-invasive marker of steatosis, has gained acceptance in clinical research for tracking changes in hepatic fat content.^24^

Comparative studies have shown that newer agents, such as GLP-1 receptor agonists (e.g., semaglutide), may achieve greater reductions in hepatic fat. However, the affordability and accessibility of pioglitazone make it a pragmatic choice in resource-limited settings, particularly for patients with comorbid metabolic syndrome.

Moreover, sustained Pioglitazone therapy can reduce the proportion of patients classified as “at-risk MASH” by the FAST™ score. While the magnitude of improvement may be modest compared to newer agents, the cost-effectiveness and dual metabolic-hepatic benefits of Pioglitazone position it as a valuable option for a broader population. In summary, this study reinforces the role of non-invasive markers, including LSM, CAP, and FAST™, in monitoring therapeutic responses. The integration of these markers into routine practice has the potential to reduce reliance on liver biopsies, which are invasive, costly, and prone to sampling error. This aligns with recommendations from recent guidelines advocating the use of non-invasive tools in both clinical and research settings.

The main limitations of this study were its retrospective design and relatively small sample size, which may limit the generalizability of the findings. Additionally, the absence of histological data restricts the ability to confirm changes in fibrosis or steatosis at a microscopic level. However, the use of VCTE is well validated to establish liver fibrosis evaluation in MASLD. Another limitation is the absence of a control group. However, the use of each patient as their own control in a paired before-after design allowed us to detect within-subject changes attributable to long-term pioglitazone therapy. While this design limits causal inference, it offers valuable real-world insights into treatment effectiveness in clinical practice. Future studies should incorporate liver biopsies or emerging imaging techniques to validate non-invasive markers and provide a more comprehensive understanding of treatment effects.

Second, the variability in treatment duration (1–10 years) introduces heterogeneity in the dataset. While the grouping of patients into short- and long-term treatment cohorts partially addresses this issue, a more uniform follow-up period would strengthen the robustness of the findings.

This supports Pioglitazone as an accessible and effective treatment choice for MASLD, especially in settings where the cost is a limiting factor for newer therapies. Improvement in liver enzymes, non-invasive scores, and hepatic fat, as evidenced by this study, propels its value in managing both hepatic and metabolic components of disease. However, further research is needed to refine its clinical role and explore its utility in combination with other therapies.

## Ethics approval statement

The Ethics Committee of the Hospital das Clínicas da Faculdade de Medicina da Universidade de São Paulo has approved this study (CAAE 80,344,824.9.1001.0068 and CAPPESQ 25,399). The protocol followed the 1975 Declaration of Helsinki.

## Availability of data and materials

The datasets analyzed in the current investigation are accessible from the corresponding author upon reasonable request.

## CRediT authorship contribution statement

**Isabel Veloso Alves Pereira:** Writing – original draft, Formal analysis, Methodology. **Ana Beatriz Souza de Oliveira:** Writing – original draft. **Patricia Momoyo Yoshimura Zitelli:** Resources. **Lais Arrivabene Barbieri:** Resources. **Ana Carolina Cardoso:** Resources. **Mísia Joyner de Sousa Dias Monteiro:** Resources. **Juliana Souza de Oliveira:** Resources. **José Tadeu Stefano:** Writing – review & editing. **Renato Altikes:** Resources. **Ana Luiza Gomes Reis:** Resources. **Cláudio de Figueiredo Mendes:** Resources. **Claudia Couto:** Resources, Writing – review & editing. **Nathalie C. Leite:** Resources. **Cristiane A. Villela-Nogueira:** Resources, Writing – review & editing. **Claudia P. Oliveira:** Resources, Writing – review & editing, Supervision. **Mário G Pessoa:** Resources, Writing – review & editing, Supervision.

## Declaration of competing interest

The authors declare no conflicts of interest.
